# Progress in Flexible Electronic Textile for Heating Application: A Critical Review

**DOI:** 10.3390/ma14216540

**Published:** 2021-10-30

**Authors:** Md. Reazuddin Repon, Daiva Mikučionienė

**Affiliations:** Department of Production Engineering, Faculty of Mechanical Engineering and Design, Kaunas University of Technology, Studentu 56, LT-51424 Kaunas, Lithuania; daiva.mikucioniene@ktu.lt

**Keywords:** conductivity, heating element, knitting, metal fibre, smart textiles

## Abstract

Intelligent textiles are predicted to see a ‘surprising’ development in the future. The consequence of this revived interest has been the growth of industrial goods and the improvement of innovative methods for the incorporation of electrical features into textiles materials. Conductive textiles comprise conductive fibres, yarns, fabrics, and finished goods produced using them. Present perspectives to manufacture electrically conductive threads containing conductive substrates, metal wires, metallic yarns, and intrinsically conductive polymers. This analysis concentrates on the latest developments of electro-conductivity in the area of smart textiles and heeds especially to materials and their assembling processes. The aim of this work is to illustrate a potential trade-off between versatility, ergonomics, low energy utilization, integration, and heating properties.

## 1. Introduction

With the progression of electronic device miniaturization and the Internet of Things (IoT), the scope of flexible wearable electronics has been increasing in our quotidian appliances. For the successful construction of flexible electronics, textiles fibrous materials have received tremendous attention because of their excellent deformability, soft feel, comfort, lightness, good absorption, and moistures properties. In general, textiles are used for clothing purposes and with the rapid growth of advanced manufacturing strategies, fibrous textiles are now acquainted as an ideal material for electronic device engineering and fabrication. The electronic textile (e-textile) can provide information that can effectively respond to and adjust actions, capable of sensing external conditions or stimuli. The stimuli can be thermal, mechanical, chemical, electrical, magnetic, optical, etc. [1,2,3,4,5,6]. According to the applications, smart textiles can be classified into three sorts: the first generation of smart textiles incorporating sensors, which can track or stimulate environmental changes is referred to as passive e-textiles; the second generation includes textiles containing sensors and actuators that give the capability to recognize and actuate or passage a part of their environment (chromatic materials, shape memory materials, phase change materials, hydrogels and membranes), which are defined as active e-textiles; lastly, the third generation of smart textiles, which can feel, respond, and accept peripheral circumstances or stimuli (space suits, thermoregulating clothing, health monitoring apparel), includes sophisticated or very e-textiles [7,8,9,10,11].

The materials, related to textiles, show conductivity or work on an electronic or computational purpose, referred to as conductive textiles, and these are used for an ample variety of textile fibre-based goods with certain electrical conductivities that vary widely [12]. Conductive fibres, yarns, fabrics, and also garments are included in conductive textiles [1]. They are required even for smart textiles to work. Their value determines smart textiles’ durability, launderability, reusability, and fibrous efficiency [13]. For antistatic applications, electromagnetic interference shielding (EMI), electronic applications, infrared absorption or protective clothing in dangerous areas, filters, de-electrifying coatings and anti-electrostatic and heating purposes, conductive fabrics have received increased interest. This is often largely because they are desirably versatile and lightweight [14,15,16,17,18,19,20]. Important advantages are gained by the textile industry within the fields of intelligent and multifunctional textile products, particularly in advanced fibres, yarns, and fabrics. Additionally, industrial materials such as sensors, electrostatic discharge, electromagnetic interference shielding, dust- and germ-free clothing, monitoring, data transfer in clothing, etc. are increasingly rising in demand for fabrics (eventually, fibres and yarns with improved electrical conductivity) [21,22]. It is possible to classify conductive fibres into two groups: those that are normally conductive, and others that are specifically treated for conductive formation. Electrically conductive metals—for example, ferrous alloys, nickel, stainless steel, titanium, aluminium, copper, and carbon—are formed from naturally conductive fibres or metallic fibres. Using coating fibres by metals, conductive polymers, galvanic substances, or metal salts such as copper sulphide and copper iodide, electrically conductive fibres can also be made. Another method comprises the preparation of polymer fibre, whose chemical configuration itself guarantees superior conductivity or the application of conductive bi-component fibres.

**Figure 1:**
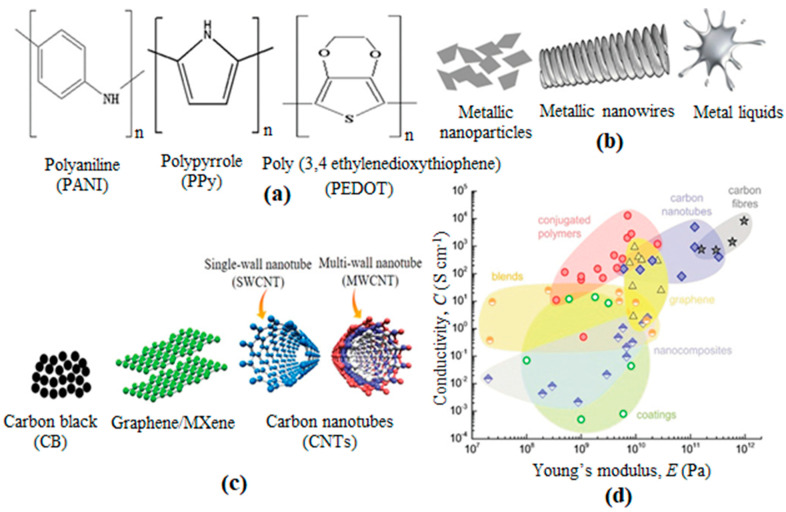
(**a**) Polymeric, (**b**) metallic, and (**c**) carbon-based electroactive materials for e-textiles. (**d**) Electrical conductivity vs. Young’s modulus of different electroactive fibres based on CNTs (blue diamonds), carbon fibers (gray stars), ICPs (red circles), blends of conjugated and insulating polymers (orange/white circles), graphene (yellow triangles), nanocomposites of CB (blue/white diamonds), CNTs or graphene embedded in an insulating polymer matrix and (green/white circles) coatings of textile fibers with ICPs, CNTs, or graphene [27].

Conductive fibres may be manufactured in filament or staple lengths to produce yarns with varying degrees of conductivity and might be integrated with conventional fibres. Without greatly altering the existing substrate properties, conductive coatings can convert substrates into electrically conductive materials. It may be implemented to the outward of fibre, yarn, or fabric via methods comprising electroless plating, evaporative deposition, sputtering, and conductive polymer coating. There is an alternative opportunity to develop conductive textiles by printing with ink that is conductive. Conductive inks are used to print patterns on fabric and those prints show electrical activity. To produce conductive inks, metals such as copper, silver, gold, carbon, and nickel are injected to conventional printing inks. Different external factors such as strain, torsion, pH, and humidity may be responsible for altering the conductivity of textile materials. For multiple novel applications, the resulting conductive textile is acceptable. Classically used electroactive materials and their properties for e-textiles are indicated in Table 1. Some chemical structures of polymeric, metallic, and carbon-based electroactive materials for e-textiles are presented in Figure 1 and electrical conductivity vs. Young’s modulus of different electroactive fibres as well.

**Table 1:** Typically used electroactive materials and their properties for e-textiles.

Electroactive Materials	Group of Electroactive Materials	Limit of Electrical Properties	Strengths and Weaknesses	Refs.
Metallic flakes/nanoparticles/nanowires (e.g., Cu/Ag/AgNWs/Au/Ni/Al)	Metal and its derivatives	≈10^4^–6.3 × 10^7^ Sm^−1^	Extremely conductive Resistant against air ageing Inflexible Less comfort	[23]
PANI/PPy/PEDOT: PSS/PhT	Intrinsically conducting polymers (ICPs)	≈10–1.7 × 10^−3^ Sm^−1^	Less cost and density Non-resistant to air ageing	[24]
CB/CNF/GO/rGO/MXene/SWCNT/MWCNT	Carbonaceous materials	≈10^2^–10^9^ Sm^−1^	Highly conductive and stretchable Time-consuming process	[25,26]

Note: Cu = copper; Ag = silver; AgNWs = silver nanowires; Au = gold; Ni = nickel; Al = aluminium; PANI = polyaniline; PPy = polypyrrole; PEDOT: PSS = poly(3,4-ethylenedioxythiophene) polystyrene sulfonate; PhT = triethoxy (phenyl); CB = carbon black; CNF = carbon nanofibres; GO = graphene oxide; rGO = reduced graphene oxide; SWCNT = single-walled carbon nanotube; MWCNT = multi-walled carbon nanotube.

## 2. Conductive Textile Architectures

### 2.1. Conductive Fibre/Yarns

**Figure 2:**
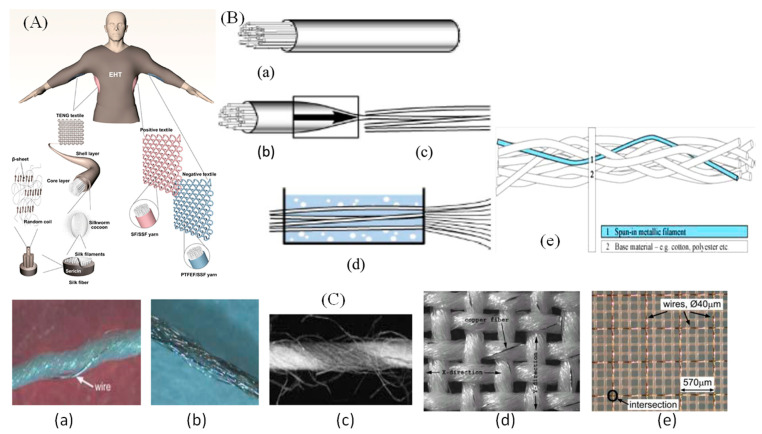
(**A**). Ultrastable and high-performance silk energy harvesting textiles; (**B**). (**a**) Iron tube; (**b**) reducing diameter; (**c**) tube building; (**d**) forming fibres and (**e**) conductive fibre diagram wrapped with the typical fibres; (**C**). (**a**) Twisted metal wire; (**b**) coated metal; (**c**) metal multi-filaments; (**d**) copper-polyester twisted yarn and (**e**) base fabric with embedded copper wire [10,40,41].

Some specific fibres that are conductive electrically are remarkable in textile history. A fibre can be described as a fine, flexible structure that has a high length-to-width ratio [28]. A fibre having an electro-conductive part can be described as conductive fibre. Thick copper wires or metal nails are electro-conductive, but they cannot be defined as fibre, since they are neither fine nor flexible. However, polymer fibre with a silver coating or fine copper wire can be identified as conductive fibres [20]. Wire drawing, a mechanical method of processing, is the traditional process of creating metal fibres. The numerous drawing measures, called coarse, medium, fine, and carding train, characterize this method (Figure 2) [10]. The drawing dies are composed of a steel mount with a centre made from ceramics, carbide, or diamond that is used for drawing the fibre. Depending on the material, the opening diameter of the metal wire fluctuates. It is typically 8 mm for copper, whereas it is 5 mm for iron. The wire is recycled at temperatures between 600 and 900 °C following drawing. They are eventually quenched. On a rotating wire drawing cylinder, the fine metal wire is then wrapped [29]. Metal monofilaments that can be intermingled with all kinds of fibres or can be employed without changing the direction in which weaving and knitting have been discovered. Significantly, there are different electrical characteristics in relation to the material used [30]. The products range from filaments made of copper (Cu) and silver-plated copper (Cu/Ag), brass (Ms) and silver-plated brass (Ms/Ag), and aluminium (Al) and copper-clad aluminium (CCA) filaments. Metal monofilaments that are inserted into base yarns such as cotton, polyester, polyamides, and aramids are specially manufactured by another company. A standard conductive yarn with base fibres and a metal monofilament twisted around them is shown in Figure 2. There, Shieldex Nylon 66 threads were used, which are coated as a base material with a thin silver layer [10]. The benefit of coatings is that they are appropriate for numerous categories of fibre and construct good conductivity without drastically modifying established main features, for example, density, flexibility, and handling. The adhesiveness of the metal and fibres plus the resistance of corrosion can, however, create problems.

Coatings may be functional to the outer part of fibres, yarns, even fabrics. Coatings on the conductive polymeric textile are carried out by sputtering, electroless plating, and deposition of vapour. In order to prepare conductive textiles, metal fibres are combined with traditional fibres during spinning [4,31,32]. During weaving and knitting, the processing of these yarns is difficult, consequential in fabrics with movable textile properties [16,33]. By coating CPs, such shortcomings connected with the processability and poor textile characteristics have been effectively solved [34]. PPy has been generally practiced by the CPs on account of its high conductivity, low toxicity, and high ecological constancy. It is presented in a system to produce fibres with diverse material layers and structures. The manufacturing process is based on the traditional fibre-processing based on the preform, easily generating kilometres of usable fibre during the process. Another important task is to produce a transistor using the crossing yarns [35,36].

Additionally, the carbon nanotube fibre is manufactured on the basis of tiny carbon nanotubes by means of the wet spinning method [37]. The groundwork and features of both conductive polymer-based fibres and nanocomposite fibres on a carbon nanotube basis are described in another paper [38]. Through melt spinning, carbon nanotubes containing conductive fine fibres were geared up with polyester, polyamide 66, and polypropylene. Here, the results showed that by adding the amount of CNT, the electrical conductivity was amplified [39].

### 2.2. Conductive Fabrics

**Figure 3:**
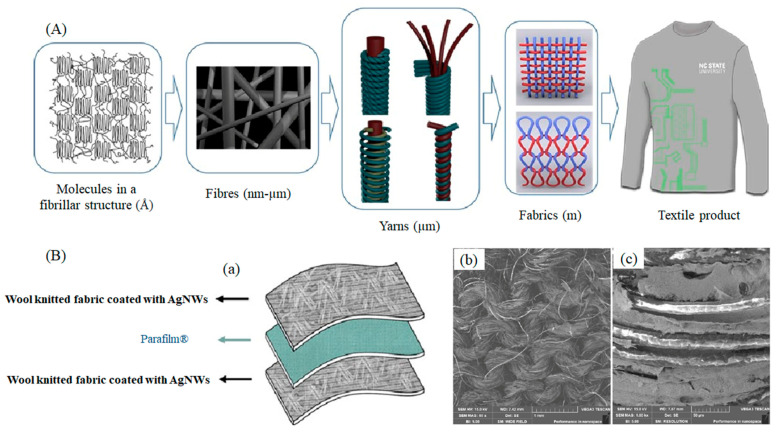
(**A**). Electrically conductive coatings for fibre-based e-textiles; (**B**). (**a**) Silver nanowire coated knitted wool fabrics for wearable electronic applications, SEM images of conductive knitting fabric after coating and washing with distilled water under (**b**) 60× and (**c**) 1000× magnifications [28,46].

Conductive textile has been gained for multi-purpose and multi-directional applications by numerous means, for instance, chemical coating, metallisation, electroless deposition, metallic yarns insertion, and thin-layer plating that holds conductive fillers, for example, CNT and carbon black particles [42,43,44,45]. By utilizing weaving, knitting, and embroidery or nonwoven manufacturing methods, the function of electronics can be incorporated into the textile products. However, it is a complex and hardly ever uniform method to incorporate conductive yarns into an arrangement as it is necessary to make sure that the properties of the conductive fabric are suitable for wearing rather than stiff and inflexible. Various kinds of threads can be utilized to launch conductivity (Figure 2). Woven fabrics can include a multiplex network with various conducting and non-conducting components that can be used as complicated electrical circuits and are designed to provide several layers and spaces to accommodate electronic devices. A simple woven fabric containing polyester yarns twisted with copper thread has been developed by researchers at ETH. It is made of 42 μm diameter woven polyester monofilament yarn and 50 ± 8 μm diameter copper alloy wires. A polyurethane varnish is used as insulation material to coat each copper wire. The copper wire grid has a separation of 570 μm in the textile structure. Electrically conductive coatings for fibre-based e-textiles and silver nanowire coated knitted wool fabrics for wearable electronic applications are exhibited in Figure 3.

Feratec^®^ (Manufacturer: Baltex; Ilkeston, UK) can primarily be used for the specific heating and electro-magnetic shielding purposes. Here, knitting technology has been used to integrate metal wires. Metallized woven nylon fabrics have also been manufactured in various shapes and profiles. In order to produce metallized woven fabrics, silver, copper, and a copper–nickel combination are used as the metal. The conductive yarns—for example, copper thread, silver-plated, and polyester-coated—are used in manufacturing electronic fabrics, electronic conductors, textile operating panels, and micro-sensors. Conductive fabric can also be accomplished using the embroidery method by adding a conductive material to a ground arrangement. Stitching patterns that can identify circuit traces, connection pads, or sensing surfaces built with conventional CAD circuit layout tools have been discovered (Figure 2) [47]. High sensitivity to its elongation is shown by the surface resistance of PPy conductive fabric. By changing the resistance and anisotropic structure of the fabric, body joint motion can be identified [48,49,50].

## 3. Thermoregulation for Conductive Textiles

Textile materials are considered as ideal insulators and hold wide varieties of thermal conductivity at their pristine state. Conductive textile uses environmentally friendly electric energy that provides electrical energy by setting heating elements into the clothing [51]. The thermal conductivity of several materials is indicated in Table 2. In order to explain the heat generation phenomenon, Joule’s law is used, wherein heat is generated by an electrical current passing through a heat-generating conductor inserted into the fabric. The power supply, flexible heating device, clothing, safety protection elements, and temperature control module are key elements of composing conductive heating textiles. Joule’s heating principle describes the heat generation; the power of heating (*P*) is related to the resistance (*R*) and electric current (*I*) of the conductor and is calculated by Equation (1).

(1) $$P=I^{2}R$$

When a resistor is connected to an external power source, it will produce heat. According to Joule’s effect, the conductive textile material can be used as a resistor with a confirmed degree of conductivity for heat generation. To effectively affect Joule’s heat generation theory, an electrical conductor necessitates a reasonable electrical resistivity. It is not possible to produce enough heat by a low-resistivity highly conductive material while passing a current through it. In order to produce resistive yarns or fabrics of conductive composite for heat generation, metal, a good conductive source, is then mixed with insulating textile fibres in a variety of ways [52,53]. The electrical conductivity, σ, is calculated by Equation (2) and the resistance, *R*, is calculated by Equation (3).

(2) $$\sigma =\frac{1}{\rho }$$

and

(3) $$R=\rho \frac{l}{A}$$

Thermal conductivity, *Κ*, is the material’s property that indicates the ability to conduct heat and is calculated by Equation (4).

(4) $$H=\frac{\Delta Q}{\Delta t}=KA\frac{\Delta T}{l}$$

where ΔQ/Δt is the rate of heat transfer, ρ is the electrical resistivity, *A* is the surface area, and *l* is the length.

The electrical and thermal energy sources are responsible for heat generation on textile materials. A suitable temperature gradient is maintained by textile heated clothing between the body and environment. It is possible to gain the necessary temperature gradient by either passive or active clothing. Where a high amount of work is needed, passive clothing is not sufficient. It impedes the wearer’s ease as it contains some layers. In that case, active clothing can be an alternative. Graphite, metal, conductive rubbers, and water-like elements have been used before in active clothing to generate heat.

## 4. Roadmap towards Heating Textile Devices

In two ways, heated textiles can be produced: one is manufacturing a piece of fabric and adding an electronic mechanism into it, and another way is manufacturing a yarn with electronic characteristics and producing textile products [54]. In heating clothing and gloves, electrical wires were used. The gloves should be used with an outward cape-leather to keep away electric wires from skin contact [55]. In World War II, metallic wires were first used in textile clothing [56]. Rather than metallic wires, more advanced conductive yarns are fashioned, currently, containing the features of textile yarns [33]. Nonwoven, knitted, woven, and embroidery fabrics can be used to fabricate heating products. Due to high resistivity, heating elements containing nonwoven fabric have confirmed limited utilization. Alternatively, woven fabric marks lower resistance than knitted fabric in the same dimensional heating area because of the structure [57,58]. A multifilament carbon blended stainless steel yarn is generated with moderate resistance for an acceptable heat generation application [4]. This kind of electro-conductive yarn has been found to exhibit especially brittle and weak bending features which are not appropriate for textile applications [59,60]. A conductive textile was first recorded by De Rossi for measuring strain and temperature [61]. By coating polypyrrole on a Lycra fabric, the sensing fabric was fabricated and demanded to demonstrate temperature sensitivity, comparable to that of ceramic thermistors. However, in order to use it in a functional environment, they have not offered any additional description. Another significant drawback was that the fabric was so particularly susceptible to a strain that could also be a major cause of strain, well in a complex setting. This may be the main source of unwanted art-crafts during temperature management. To produce active clothing, external devices or heaters were used, though there were some limitations of using heaters: clothing weight up, structure rigidity, and sweat extraction. Heating small patches can be without problems joined in active clothing at several locations by stitching. Basically, a patch of heating is composed of four elements: a carrier, a heating material, a bus bar, and a power source. Many of the incidents recorded in recent years are attributed to aircraft caused by ice accumulation [62,63]. Heated textiles can also be exercised as an anti-freezing mediator in the aircraft industry to prevent ice accumulation on the aircraft wings.

Heating textile has therapeutic advantages, providing heat treatment against persistent pain [64]. As the surface of the heating textile remains in contact with skin, it shows effective results in thermotherapy. This textile is used in a particular region and the application of heat increases blood flow, reduces inflammation and pain, and is also used for joint injuries [65,66,67]. To inform patients of their pathological conditions, heating textiles can play a role as thermographs. It can monitor vascular, dermatological, and rheumatic abnormalities and investigate breast cancer [68]. In that case, thermocouples and thermistors are used as contact sensors and can be inserted into textiles without making them bulky [69]. In the next section, the recent progress in different electroactive materials coated heating textile is comprehensively discussed.

## 5. Advanced Heating Textiles and Their Performances

### 5.1. Metal-Based Heating Textiles

Metals are highly electro-conductive and have ideal electrical properties as well. That is why metal-integrated textiles are conductive and can be used for heating purposes. Frank Hewitt manufactured heating fabric and heating pads where heat was produced by electric current flow through a metal wire that was inserted into the fabric or pad [70,71,72]. Metal wires can be woven or knitted into the heating textile. The heating fabrics which have plain and interlocking knitted structures show maximum equilibrium temperature at the voltage of 3 V and stainless steel yarns are integrated as heating elements into the knitted fabrics where silver-plated yarn is braided on both sides of the fabrics [73]. A study shows that plain woven fabric which contains silver filament or silver-plated yarns has a great positive linear correlation between power and temperature. This relation improves the design of fabric manufacturing by analyzing the physical properties of heating textiles [57]. To prepare metal-based conductive heating textile, metal nanomaterials can be coated on the fabric surface. Compared to CNT-coated cotton fabric, AgNW-coated cotton fabric shows a better heating effect. The study shows that at the voltage of 0.9 V and 1.2 V AgNW-coated cotton fabric can reach 38 °C and 53 °C, respectively, whereas to obtain the same heating effect for CNT-coated cotton fabric, the voltage needs to be raised up to 12 V [74,75].

**Figure 4:**
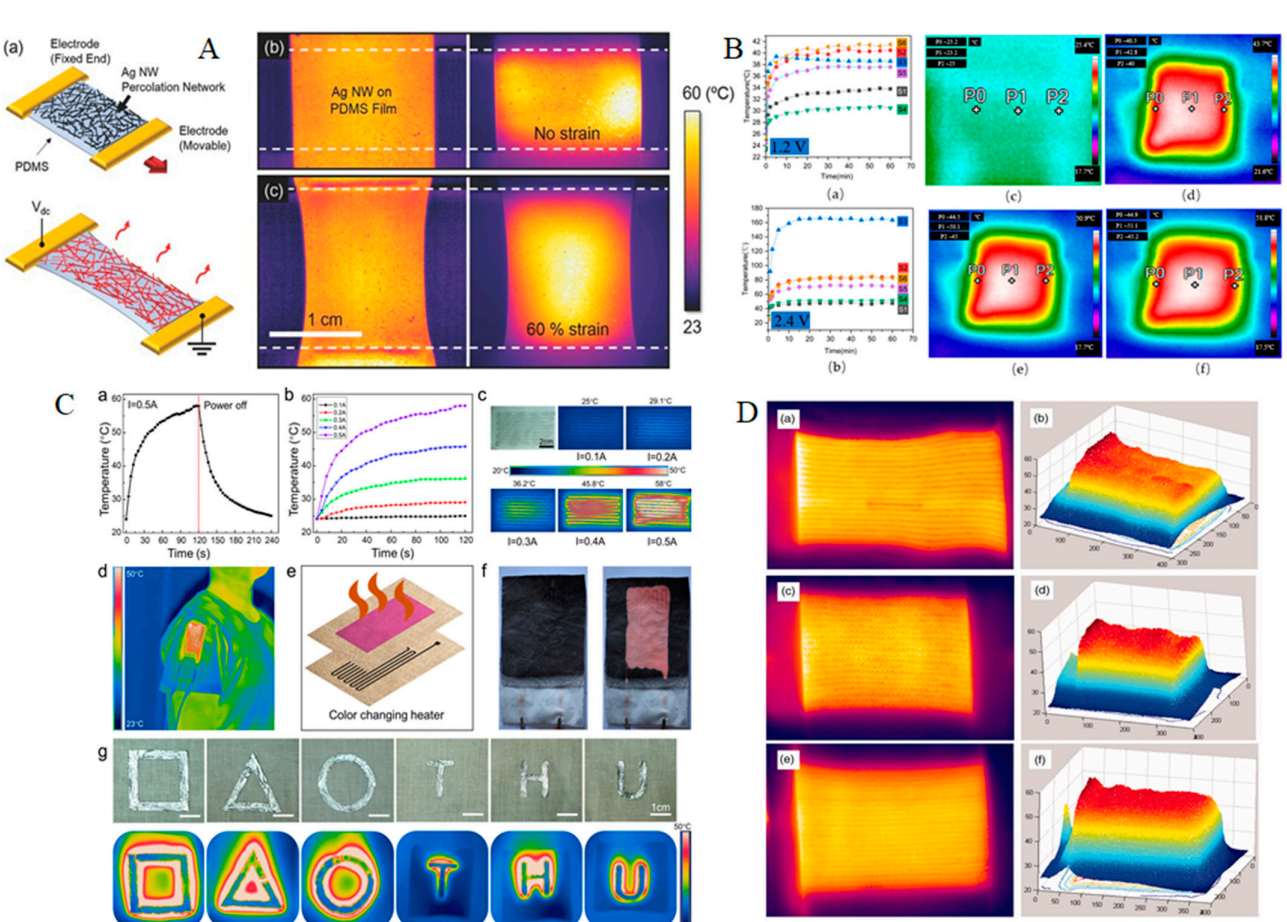
(**A**) Highly stretchable and transparent heater. (**a**) Schematic illustration of the stretchable and transparent heater composed of AgNW percolation network on PDMS film; (**b**,**c**) pseudocolor image at room temperature (left) and infrared camera thermal image (right) of a Ag NW/PDMS stretchable and transparent heater operating at 60 °C with (**b**) no strain and (**c**) at 60% strain condition [79]; (**B**) sample temperature change with time of S1–S6 under (**a**) 1.2 V and (**b**) 2.4 V and thermal images of S4 (**c**) before and (**d**) after 1 min, (**e**) after 30 min, and (**f**) after 1 h of application of a voltage of 2.4 V [78]; (**C**) thermal management device printed on woven cotton fabrics. (**a**) Temperature curve of the serpentine Cu-EGaIn conductors during heating/cooling process under input current of 0.5 A. (**b**) Temperature curve of the serpentine Cu-EGaIn conductors during the heating process under various input currents. (**c**) Picture of the thermal management device and its infrared temperature distribution images at 120 s under various input currents. (**d**) Infrared temperature distribution image of the thermal management device sewn on a T-shirt. (**e**) Structure diagram of the woven cotton fabric with color changing pigment. (**f**) Thermochromic display of the serpentine Cu-EGaIn conductors. (**g**) Infrared temperature distribution image of multiple Cu-EGaIn patterns heated by the electromagnetic heating coil [80]; (**D**) infrared images of (**a**) PSF, (**c**) RSF and (**e**) ILK and three-dimensional images of (**b**) PSF, (**d**) RSF and (**f**) ILK at 4 V [76].

Liu et al. [76] knitted plain, rib, and interlock structures using silver-plated yarn and polyester staple yarn (Figure 4D). In the research, aging tests were carried out, and under 100 °C temperature, the silver-plated yarn was hardly affected by time and the aging temperature. A strong linear correlation was found between the power consumption density and the maximum equilibrium temperature of three knitted fabrics in the study. Another research work was carried out to test the electro-thermal stability of silver yarn or silver-coated yarn by performing an oven aging test; the results found better electrical resistivity of silver yarn by showing a strong linear density. At a voltage of 9 V, that resistivity made the samples capable of obtaining higher temperatures [77,78]. Kexia et al. [78] showed the relation of temperature and the resistance of wool and silver yarn made conductive electro-thermal knitted fabric (Figure 4B). At a voltage of 2.4 V, the conducting heating fabric showed a better result which had the double needle bed knitting structure of 1 × 1 rib. Hong et al. [79] embedded AgNWs to polydimethylsiloxane (PDMS) films (Figure 4A). These conductive films showed extraordinary electrical conductivity that could respond to thermal properties quickly by generating Joule heating (Figure 4A). Guo et al. [80] used the roller printing method to create highly conductive wearable electronics for smart fabrics depending on the adhesion variance of semiliquid metal (Cu-EGaIn, eutectic gallium-indium combined with copper microparticles) on cotton fabrics and PVAC glue. The adhesive effect with the Cu-EGaIn mixture is determined by the surface topography and chemical interaction of textiles and PVAC glue, according to the findings. The electromechanical stability of the manufactured lines on fabrics was proved in the electric testing. To demonstrate practical applications in the method, a number of smart fabrics were constructed, including an interactive circuit, stretchy light-emitting diode array, and thermal management device with benefits of easy operation, low cost, and large-area fabrication (Figure 4C). Repon et al. [81] investigated heat generation in compression supports using Ag-coated PA-based electro-conductive knitted textiles. They created compression knitted constructions with integrated electro-conductive yarns and studied heat generation characteristics and temperature variations over time and under stretch to induce compression. Silver-coated PA yarn with linear densities of 66 tex and 235 tex was used to create combined half-Milano rib structured knitted fabrics. The summary of metal-coated heating textiles is shown in Table 3.

**Table 3:** Summary of metal-coated heating textile.

Electroactive Materials	Type of Textile	Temperature Range (°C)	Voltage Range (V)	Electrical Properties	Refs.
AgNWs	Cotton/Polyurethane Core-Spun Yarn (CPY)	≈25–100	2–6	≈36 Ωsq^−1^	[82]
AgNWs	Nylon	≈30–140	2–10	30 Ωsq^−1^	[79]
AgNWs Ink	Polyester (PET)	≈20–100	3–7	≈10 Ωsq^−1^	[83]
AgNFs and PtNFs	Silk fabric (SF)	41.3–99	3–8	25 Ωsq^−1^	[84]
AgNFs	Silk fibroin (SF)	≈28–106.2	0.5–4.5	12 Ωsq^−1^	[85]
AgMFs	-	27.3–209.4	0–1.6	˂0.2 Ωsq^−1^	[86]
AgFDs	Polystyrene film	52.3–180<	1–4	0.048 Ωsq^−1^	[87]
AgNWs	PVA Film	≈45–74	3–5	20 Ωsq^−1^	[67]
AgNWs	Polyester (PET) and polydimethylsiloxane (PDMS)	≈30–160	5–25	≈0.5 Ωsq^−1^	[88]
AgNWs	Polydimethylsiloxane (PDMS)	≈50–160	1–2	0.25 Ωsq^−1^	[89]
CuNWs	Polyurethane (PU)	46–102	3–7	4.7 Ωsq^−1^	[90]
Cu-Ni NWs	Poly (ethylene terephthalate) (PET)	20–106	3–15	300 Ωsq^−1^	[91]
AgNWs	Elastomer	≈40	0.5–1.0	≈0.8 Ω	[66]
AgNPs	Cotton fabric	≈34–98	1–5	0.26 Ωsq^−1^	[92]
AgNW/PEDOT: PSS	Silk yarn	25–64	2–3	≈320 S/cm	[93]
CuZr	Metallic glasses	180	7	3.8 Ωsq^−1^	[65]
AgNWs	Cotton fabric	≈42	1.5	2.2 Ωsq^−1^	[94]
Cu filament	PET-Cu braided fabric	≈89	5	2.428 Ω/m	[95]
Stainless steel yarns	Polyester knitting fabric	≈60	12	-	[54]
CuNWs	PET fibres	57	3	-	[96]
Silver-plated yarn	Polyester staple yarn	4	70	-	[97]
Stainless steel	Cotton fabric	≈84 (plain) ≈99 (Interlock)	3	0.3 Ω cm^−1^	[73]
Stainless steel, CB	Cotton fabric	≈63	6	1.2–12 kΩ	[98]
Ag	Cotton-nylon spandex fabric	≈119	10	21 Ω mm	[99]
Ag	Polyamide	52	2.5	0.64 Ω	[81]
AgNPs	Cotton	36.5–118.7	0.5–2.0	-	[100]
CuNWs	Polyamide 6	70	1.8	0.3 Ωsq^−1^	[101]

Note: AgNWs = silver nanowires; AgNFs = silver nano-fibres; AgFDs = silver micro-fibres; CuNWs = copper nanowires; PEDOT: PSS = poly(3,4-ethylenedioxythiophene) polystyrene sulfonate; CB = carbon black; Cu = copper; Ag = silver.

### 5.2. Conductive Polymer Based Heating Textiles

**Figure 5:**
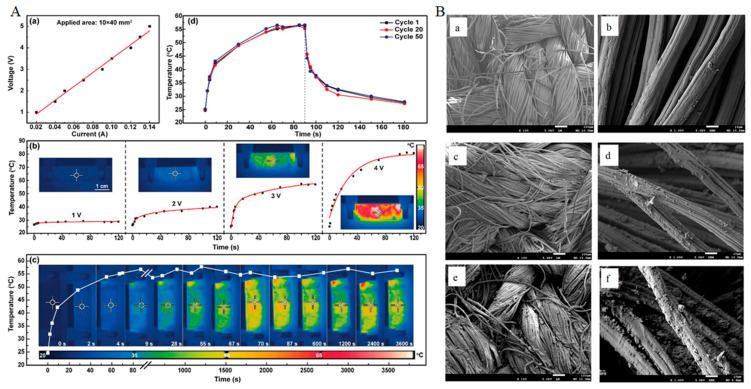
(**A**). I–V curve and (**b**) Joule heating performances of silicone-coated M-textile. (**c**) Time–temperature curve at a constant voltage of 3 V for silicone-coated M-textile. (**d**) Temperature stability of the silicone-coated M-textile in heating/cooling cycles [109]; (**B**). SEM images of CBPFs and GCBPFs ((**a**,**b**) CNC-coated bamboo pulp fabric; (**c**,**d**) G/CNC-coated fabric with lowest thermal conductivity; (**e**,**f**) G/CNC-coated fabric with highest thermal conductivity) [124].

It has been found that polymeric composites of non-metals are ideal for generating heat. The benefits of these heating yarns are found, compared with other heating materials, for their low power density, lightweight, temperature homogeneity, and their durability and fineness [102,103,104,105]. To prepare conductive composites, PPy, PANi, PTh, etc. polymers are used for coating on yarns by different systems. Polypyrrole (PPy) can be coated by chemical and electrochemical methods on nylon fabric. By the gas combustion method, PPy-coated polyester fabric can be obtained where FeCl3 is used as an oxidizing agent [106,107]. A combination of in situ polymerization and interfacial polymerization method is used to coat a thin and compact PPy layer on cotton, silk, wool, and polyester fabric and their temperature can rise to 100 °C at 6 V [108]. Wang et al. [109] fabricated highly conductive and hydrophobic textiles that showed an excellent heating performance according to Joule’s law and the conductivity of the fabric was 1000 Sm^−1^. In the experiment, silicone-coated MXene sheets that were modified by in situ polymerized polypyrrole (PPy) were deposited onto poly (ethylene terephthalate) textiles. Joule heating performances, current-voltage behaviour and temperature stability of silicone-coated M-textile and the SEM images of G/CNC-coated bamboo viscose fabrics are displayed in Figure 5.

Studies show that integrating PPy into fibres, both natural and regenerated, to form a textile composite and also cotton woven fabrics is appropriate for heat creation [110,111]. Sparavigna et al. and Macasaquit and Bina recommended that 100% PPy-coated polyester fabrics are basically functional for large extent purposes, together with medical or other applications among flexible, portable, surface-heating elements [112,113]. There is fair conductivity in PPy-coated polyester–Lycra woven composite fabrics, and efficient heat generation [114,115]. The rate of temperature change has two separate phases for all these composites, an initial sharp rise followed by a leveling-off to plateau, close to PPy-coated cotton composites [116]. It has been noted that the doping anion controls the heat and resistivity outcome of PPy composites [117]. By chronological HTHP chemical and electro-chemical polymerizations, nylon fabric with PPy coating is structured. By using a marketable battery of 3.6 V, the surface temperature of this fabric goes up rapidly to around 55 °C in 2 min and it is constant for at least ten rounds [106]. There is rational electrical stability in a PPy-coated e-glass fabric, and it is found to be efficient in generating heat. The surface temperature increases by applying an unvarying voltage through the cloth, while power consumption is found to be decreased [118]. For heat generation, PPy-coated silk composites are also arranged [119,120]. The application of voltage increases the temperature of the PPy-coated woven and non-woven fabrics and studies show the rate of increasing temperature increases exponentially. It is also noticed that the time duration of applying voltage is an important factor for increasing the temperature of the fabrics [121].

The polymer of 3, 4-ethylene dioxophene monomer (EDOT) is called polyethylene dioxophene thiophene (PEDOT). PEDOT has been used for fabricating conductive heating textiles due to its features of high conductivity, simple molecular design, and compact energy gap. In order to synthesize PEDOT with the substrate material, several techniques are used such as spraying, impregnation coating, in situ polymerization, and vapor phase polymerization. To manufacture conductive PET, Yang et al. [122] coated PET with PEDOT film applying the vapor phase polymerization process. Research shows better reusing stability and uniform thermal distribution of AgNWs/PEDOT: PSS composite film by studying its thermal response features on the basis of thermodynamic analysis, the heat capacity of substrate material [123]. The summary of polymer-coated heating textiles is stated in Table 4.

**Table 4:** Summary of polymer-coated heating textile.

Electroactive Materials	Type of Textile	Temperature Range (°C)	Voltage Range (V)	Electrical Properties	Refs.
PEDOT: PSS, rGO	Cotton fabric	70	30	150 Ω sq^−1^	[125]
PEDOT, MXene	Cotton fabric	≈193	12	3.6 Ω sq^−1^	[126]
PPy, FeCl3	Cotton fabric	≈168	5	0.37 Ω cm	[127]
PPy	PET fabric	≈110	30	1434.12 Ω sq^−1^	[121]
PPy	Cotton fabric	≈48	9	32 Ω sq^−1^	[128]
PPy, MXene, silicone	PET fabric	≈57	3	≈1000 S m^−1^	[109]
PPy	PET-lycra fabric	≈40	24	150–500 Ω sq^−1^	[115]
Polypyrrole	Nylon fabric	55	3.6	5 Ω sq^−1^	[107]
Polyethylene dioxophene thiophene	PET	43	15	52 Ω sq^−1^	[129]
PEDOT	Cotton fabric	≈44	6	41 Ω sq^−1^	[130]
PEDOT: PSS, SDS	Cotton fabric	≈99	12	1335 Scm^−1^	[131]
PEDOT: PSS, glycerol	Polyamide fabric	≈80	12	740 Ω	[129]
rGO	PET/PU fabric	≈59	30	2.0 × 10^−5^ S sq^−1^	[132]
rGO	PET fabric	≈138	14	24.7 Ω sq^−1^	[133]

Note: PPy = polypyrrole; PEDOT: PSS = poly(3,4-ethylenedioxythiophene) polystyrene sulfonate; GO = graphene oxide; rGO = reduced graphene oxide.

### 5.3. Carbon Based Heating Textiles

**Figure 6:**
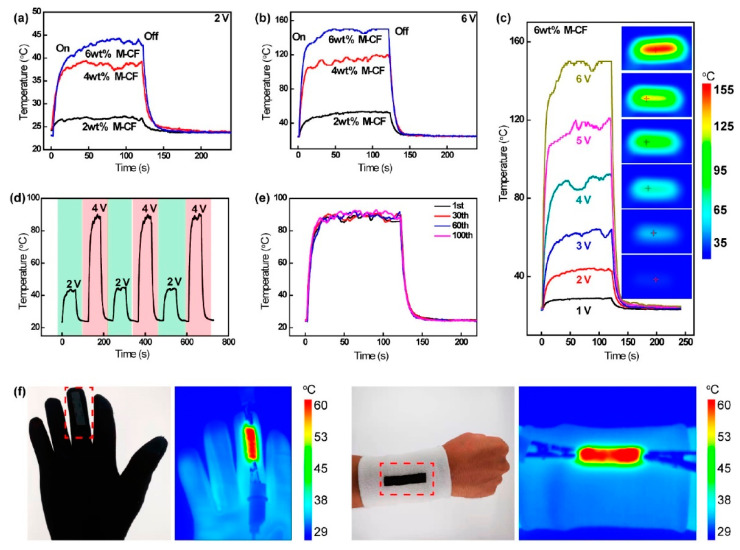
A piece of flexible MXene-decorated fabric with interwoven conductive networks for integrated Joule heating, electromagnetic interference shielding and strain sensing performances: Time–temperature curves of M-C fabrics at a voltage of (**a**) 2 and (**b**) 6 V. (**c**) Time–temperature curves of 6 wt % M-CF from 1 to 6 V. (**d**) Temperature adjustability of the 6 wt % M-CF heater. (**e**) Temperature stability of 6 wt % M-CF heater under 100 heating cycles. (**f**) Temperature distribution of the wearable 6 wt % M-CF heater attached on a glove and wrist [141].

**Figure 7:**
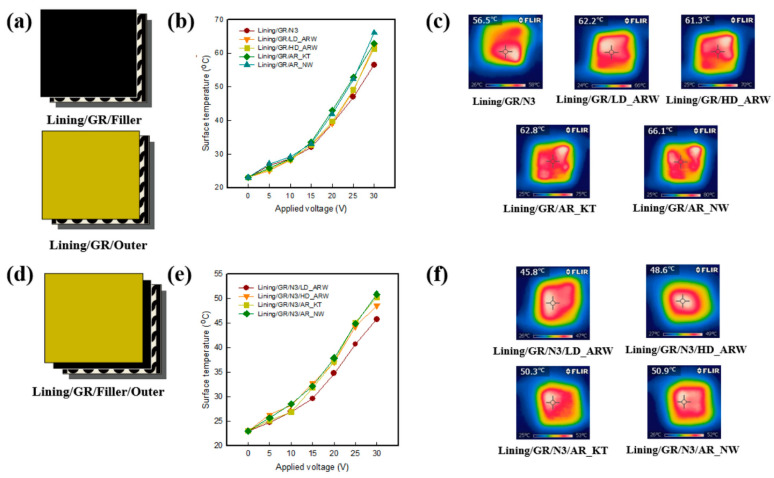
Thermal insulation property of graphene-/polymer-coated textile-based multi-layer fabric heating element with aramid fabric: (**a**,**d**) measuring layer of triple- and quadruple-layer samples. (**b**,**e**) surface temperature of triple- and quadruple-layers of samples with various applied voltages, and (**c**,**f**) IR images of each sample at 30 V [142].

Conductive materials based on carbon are a good source of heat generation. Graphene, graphite powder, and CNTs are normally attached to the surface of the fibre to make it conductive. By using recycled carbon fibre and dipping and coating CNTs with cotton fabric, where SWCNTs are used as a dispersant, the conductive heating textile can be developed [134,135]. To enhance the thermal properties of the fabric, MWCNTs are coated on the cotton fabric through the techniques of impregnation–drying coating and the improvement of thermal conductivity is found [136]. Time–temperature curves for different voltage and temperature stability of flexible MXene-decorated fabric with interwoven conductive networks for integrated Joule heater are shown in Figure 6. For heat generation application, reduced graphene oxide (rGO) nanosheet-coated cotton fabric-based films are used to fabricate electrically conductive textile [137]. Wearable electronics, specific devices for personal cooling and heating, or simply personal thermal management (PTM) devices can be developed by embedding graphene in fabrics. Using highly conductive graphene fibre with superior heating at reduced energy can readily increase stretchability and breathability [138,139,140]. Surface temperature of triple- and quadruple-layers at various applied voltages and IR images of graphene/polymer coated textile based multi-layer fabric heating element with aramid fabric are shown in Figure 7. The summary of carbon based heating textile is demonstrated in Table 5.

**Table 5:** Summary of carbonaceous heating textile.

Electroactive Materials	Type of Textile	Temperature Range (°C)	Voltage Range (V)	Electrical Properties	Refs.
rGO/PEDOT: PSS	Cotton fabric	30–70	5–30	150 Ω sq^−1^	[125]
MWCNT	Glass or poly(dimethylsiloxane) (PDMS)	100	40	172 Ω sq^−1^	[143]
MWCNTs	Cotton	≈90	10–60	1670 Ω sq^−1^	[144]
MWCNTs	Silk Fabric	≈49.1	5–25	468 Ω sq^−1^	[145]
Graphene	Polyimide	55–150	30–60	1.568 Ω sq^−1^	[146]
rGO	Polyester fabric	50–138.64	6–14	24.7 Ω sq^−1^	[133]
CB	Polyester Fabric	≈30–85	0–20	<71 Ω cm^−1^	[147]
CNT	Cotton Yarn	Max. 80	2–5	3.92 Ω cm^−1^	[148]
CC/PW	Thermoplastic Polyurethane (TPU)	≈32.5–50	2–3	374 Sm^−1^	[149]
MnO2/rGO	Cotton fabric	Max. 36	1–15	0.78 Ω	[150]
Graphene/WPU	Polyester	71.3	50	5.43 × 10^3^ Ω sq^−1^	[151]
Recycled carbon fibre	non-woven fabric	94.6	13	2.8 × 10^3^ Sm^−1^	[152]
Graphene, WPU	Aramid fabric (knit)	≈54	5	≈56 Ω	[153]
Graphene, MWCNTs	Cotton	66.2	27	29.8 Ω sq^−1^	[154]
MWCNTs	Polyester/polyurethane	56.1	5	2.66 Ω cm	[155]

Note: PEDOT: PSS = poly(3,4-ethylenedioxythiophene) polystyrene sulfonate; CB = carbon black; CNT = carbon nanotube; GO = graphene oxide; rGO = reduced graphene oxide; MWCNT = multi-walled carbon nanotube; WPU = waterborne polyurethane.

**Figure 8:**
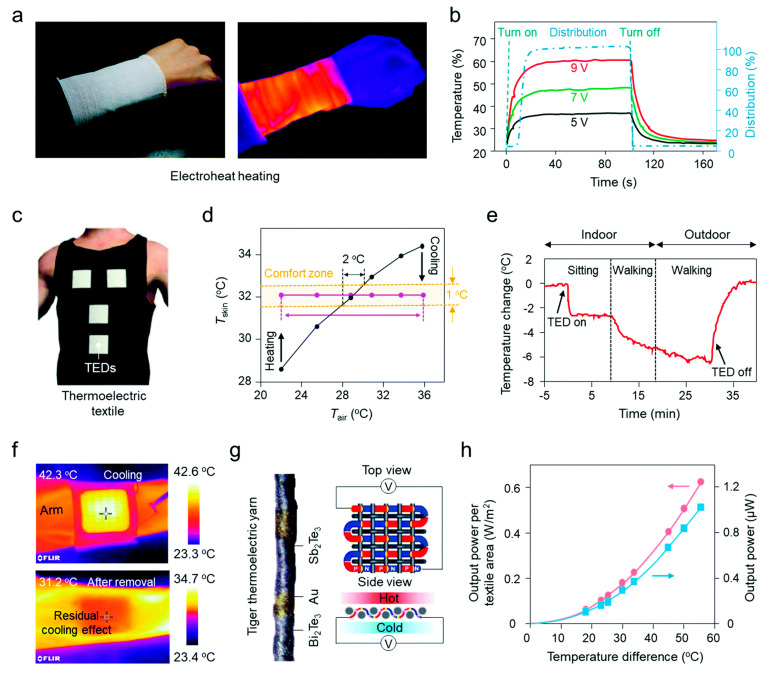
Active thermoregulation via electro-heat and thermoelectrics. (**a**) Photograph and IR image of a stretchable and energy-efficient smart Joule-heating textile. (**b**) Temperature and distribution profiles of the smart heating textile. (**c**) Smart textile with wearable thermoelectric devices (TEDs). (**d**) Skin temperature as a function of air temperature with a TED-based smart textile. (**e**) Skin temperature regulation via a TED-based smart textile under daily conditions. (**f**) IR image of a TED-based smart textile on and off the skin for temperature regulation. (**g**) Thermoelectric yarn and woven textiles for thermoregulation. (**h**) Output power of the plain-weave thermoelectric smart textile [156].

Wearable thermoelectric devices have the potential to generate electricity for on-body applications in a ubiquitous, non-intermittent, and noiseless manner. Due to its out-of-plane thermoelectric generation and strong structural conformability with fabrics, three-dimensional thermoelectric textiles (TETs) outperform other varieties in smart textiles [156,157,158,159,160,161]. Yuanyuan et al. [157] sewed carbon nanotube yarn-based segmented thermoelectric textiles on a large scale to create organic spacer fabric shaped TETs. It was discovered through a combination of finite element analysis and experimental evaluation that the fabric structure has a substantial impact on power generation. The properly constructed TET has a high output power density and is wearable and stable. Another study generated a stretchable heating carbon nanotube (CNT) fibre with good mechanical and heating capabilities based on the hierarchically helical structure of traditional thermal insulation material—wool [159]. A huge number of generated hierarchically helical voids inside and out provide good thermal insulation. It has a rapid thermal response of over 1000 °C s^−1^, a low working voltage of a few volts, and great heating stability over 5000 cycles, to name a few features. Active thermoregulation via electro-heat and thermoelectrics is illustrated in Figure 8. The applications of fibres and yarns in smart fabrics along with conductive features are mentioned in Table 6 and the coating techniques of textile yarn with intrinsically conducting polymer (ICP) are stated in Table 7.

**Table 6:** Application of fibres and yarns along with conductive features in smart fabrics.

Material	Mesh or Core	Characteristics	Advantage	Disadvantage	Resistance per Unit Length	Refs.
Copper wire/tinsel wire	Polyester, copper (tinsel)	Flattened and twisted with cotton, nylon, Nomex or Kevlar thread	Robust connection, conventional	Difficult to integrate into clothing	~21 Ω cm^−1^	[162]
Stainless steel staple fibres	Blended with polyester	Composite broken bundles (sewable)	Strength, resistance to corrosion, biologicalinertness	Difficult to attach to existing electronics components	BK 50/2 ~50 Ω cm^−1^, broken)	[47]
Aracon MCAF metal clad aramid (polymer) fibres	Kevlar	Composite core: Kevlar cladding metal: Ag, Ni, Cu, Au, Sn (24–200 fibres)	Light, flexible, stable, high temp resistance Can be soldered like normal wire	Conformability in integration with fabrics	~0.001 Ω cm^−1^	[47]
Metallic organza	Cloth	Composite fibre: Ag	Yarn level integration	Challenging connections to data acquisition	~10 Ω m^−1^	[47]
Silver thread	Fabric	Composite2 ply Ag fibre, nylon	Machine sewable	Sensitive to humidity and aging	~85 Ω ft^−1^	[163]
Strips of conductive fabric	Coated or intr. conductive fabric	Carbon based, PPy, PEDOT, PANi, metal plated (i.e., Cu, Ni)	Can be glued, sewed to other fabrics	Compatibility and specialty of connectors	Varies	[164]
Thin Kapton sheet	Kapton	Stacking of thin film layers including silicon nitride	Enables flexible electronics techniques	Cannot be machine sewn	Varies	[165]

**Table 7:** Coating techniques of textile yarn with ICP.

Coating Technique	Textile Yarn	ICP	Linear Resistivity (Conductivity)	Refs.
Solution polymerization	Wool, cotton, nylon, and polyester	PANI	23 kΩ/cm/ filament	[166]
Dipping and drying	PET	PANI	~70 Ω/cm	[11]
Dipping and drying	PET	PANI	~100 Ω/cm	[42]
Solution polymerization	Wool	PPy	4.8 kΩ/cm	[167]
Solution polymerization	Wool	PPy	~50 Ω/cm	[168]
Vapour polymerization	Wool, cotton, and nylon	PPy	0.37–3 kΩ/mm	[169]
Vapour polymerization	Wool	PPy	0.43 kΩ/mm	[170]
Vapour polymerization	Nylon-6 and polyurethane	PPy	----	[171]
Vapour polymerization and solution polymerization	Cotton and silk	PPy	6.4×10^-4^ S/cm (cotton) 3.2×10^-4^ S/cm (silk)	[172]
Dipping and drying	Silk	PEDOT: PSS	8.5 S/cm	[173]
Dipping and drying	Silk	PEDOT: PSS	2 kΩ/mm	[34]
Vapour polymerization	Viscose	PEDOT: PSS	----	[35]

Note: PANI = polyaniline; PPy = polypyrrole; PEDOT: PSS = poly(3,4-ethylenedioxythiophene) polystyrene sulfonate.

## 6. Future Perspective and Conclusions

In antiquity, conductive threads were fabricated before the discovery of electricity. With the passage of time, things change, and different innovations are carried out day by day to improve the path of conductivity in textile. Textile fibres, yarns, and fabrics are modified by using metal in the form of wires, coats, wraps, and conductive polymers intrinsically. Traditional electrical wires were initially used, but more advanced methods were then introduced. Particularly with the high expansion in wearable and electronic materials, there will be an additional drive for the enlargement of conducting paths with properties more compatible with traditional fibrous materials. Smart textiles and their applications will boom in the forthcoming in the area of textiles, electronics, and information technology with the development of advanced materials and polymers. The future possibilities for conductive material comprise health, protection, fashion, and fitness. The smart conductive textile can give protection to the firefighters and can monitor health in clinical application, use in sportswear for fitness purposes, functional clothing for fashion and non-clothing are applied for the automotive and home textile purpose.

Textile-based Joule heaters by implementing a combination of nanomaterials, fabrication tactics, and structural designs have changed the paradigm of futuristic intelligent garments and clothing systems. Structural textiles have different benefits over rigid electrical elements such as comfort, feasibility, flexibility, breathability, etc. in designing thermo-regulating devices. In spite of immense progress in textile-based Joule heaters, there still remain some challenges to overcome. It is very difficult to retain the electrical properties or constant heating functionality under mechanical deformations and wet conditions (e.g., wash/sweating). Wearable textile heaters with insufficient durability under critical environments make the acceptability of numerous prototypes questionable for large-scale applications. To keep the electrical functionality activated from creasing or washing stresses, a strong interfacial bonding (intimate contact between textiles and embedding materials) is essential. Moreover, the implementation of flexible encapsulant materials such as 3D printing bundle, fully biobased film, and ultrathin White-EVA films are expected to guarantee a promising bright future for textile-based thermoregulation in the coming decade.

Textile-based heaters open new opportunities for next-generation smart heating devices. Furthermore, problems coexist with conveniences, necessitating increased study and improved tactics to impart higher functionality, wearability, aesthetics, and washability into a textile heating device while maintaining adequate mechanical compliances. Various important understandings are still to be uncovered in order to convey the true impact of these wearable textile heaters to our society and to accelerate their large-scale production. We believe that with the continued hard work of researchers from many areas, the design, functionalities, and performance of heating textiles will be enhanced, thereby promoting the development of heating textiles toward a future.

## Figures and Tables

**Table 2:** The thermal conductivity of several materials [51].

Indicators of Thermal Conductivity of Thermal Insulators	Thermal Conductivity (λ/Wm^−1^ K^−1^)
Wood	0.17
Asbestos	0.17
Plastics	0.17
Leather	0.15
Polystyrene	0.1329
Polyacrylonitrile fibres	0.05
Nylon	0.209–0.337
Polypropylene fibres	0.22–0.30
Cellulose	0.11
Air	0.0244

## Data Availability

Data Sharing is not applicable.
